# Application of molecular cytogenetic techniques to clarify apparently balanced complex chromosomal rearrangements in two patients with an abnormal phenotype: case report

**DOI:** 10.1186/1755-8166-2-15

**Published:** 2009-07-13

**Authors:** Paula JP de Vree, Marleen EH Simon, Marieke F van Dooren, Gerda HT Stoevelaar, José TW Hilkmann, Michel A Rongen, Gido CM Huijbregts, Annemieke JMH Verkerk, Pino J Poddighe

**Affiliations:** 1 Department of Clinical Genetics, Erasmus MC, Rotterdam, the Netherlands; 2 Department of Bioinformatics, Erasmus MC, Rotterdam, the Netherlands

## Abstract

**Background:**

Complex chromosomal rearrangements (CCR) are rare cytogenetic findings that are difficult to karyotype by conventional cytogenetic analysis partially because of the relative low resolution of this technique. High resolution genotyping is necessary in order to identify cryptic imbalances, for instance near the multiple breakpoints, to explain the abnormal phenotype in these patients. We applied several molecular techniques to elucidate the complexity of the CCRs of two adult patients with abnormal phenotypes.

**Results:**

Multicolour fluorescence in situ hybridization (M-FISH) showed that in patient 1 the chromosomes 1, 10, 15 and 18 were involved in the rearrangement whereas for patient 2 the chromosomes 5, 9, 11 and 13 were involved. A 250 k Nsp1 SNP-array analysis uncovered a deletion in chromosome region 10p13 for patient 1, harbouring 17 genes, while patient 2 showed no pathogenic gains or losses. Additional FISH analysis with locus specific BAC-probes was performed, leading to the identification of cryptic interstitial structural rearrangements in both patients.

**Conclusion:**

Application of M-FISH and SNP-array analysis to apparently balanced CCRs is useful to delineate the complex chromosomal rearrangement in detail. However, it does not always identify cryptic imbalances as an explanation for the abnormal phenotype in patients with a CCR.

## Background

Complex chromosomal rearrangements (CCR) are defined as structural abnormalities involving more than two breakpoints and the exchange of genetic material between two or more chromosomes [1]. They can occur in patients who are mentally retarded or have multiple congenital abnormalities [2,3] or in phenotypically normal individuals who are ascertained through the birth of a malformed child or fetus, repeated abortion or reproductive problems [4-6]. Until now, more than 160 patients with a CCR are reported in literature, observed both postnatally as well as prenatally [7-11]. This number will increase since the application of molecular cytogenetic techniques on apparently balanced reciprocal translocations has revealed that more cryptic rearrangements, with or without imbalance, can be found [12-17]. Multicolour fluorescence in situ hybridization (M-FISH) can visualize the complexity of structural rearrangements in one single overview, sometimes undetected by conventional cytogenetics, by applying 24 distinct colours separating one chromosome from the other [18,19]. The application of molecular high resolution SNP-array analysis on DNA of patients with an abnormal phenotype and apparently balanced chromosome rearrangements may detect submicroscopic imbalances [20,21] that could have an association with the disease. The combination of both techniques will lead to the identification of more chromosomal breakpoints or genomic imbalances, giving more insight into the complexity of the chromosomal rearrangements.

Here we present two adult patients with an abnormal phenotype, both with a *de novo *initially apparently balanced CCR determined by GTG banding. The application of M-FISH, SNP-array and FISH analysis has clarified the CCR in more detail in order to perform a genotype-phenotype study.

## Case presentation

### Patient 1

The patient was the second child of non-consanguineous parents. He was born by caesarian section because of a high head position. His apgar score was 9 after 1 minute. Birth parameters were normal (weight 3380 grams, length 49 cm, head circumference 37 cm). There was a slight delay in early development as walking and first words began at the age of 18 months. Further speech development was slow with poor articulation. At the age of 4 years, an autistic spectrum disorder was suspected because of stereotypic movements and typical behavioral problems. Because of his mild mental retardation he attended special education. At puberty, his weight increased with 20 kg in 2 years. Autistic behavior had diminished after puberty, though he still clung to regular daily patterns. His general health was good and vision and hearing were normal.

At the age of 15 years and 5 months, his length was 183,8 cm (+1 SD), weight 112 kg (>>+2 SD), and head circumference 60,4 cm (+2,5 SD). He had a relatively large head with bitemporal narrowing and a mildly sloping forehead. His eyebrows were full and broad. His eyes were deep-set with epicanthic folds and slightly downslanting palpebral fissures. He had a bulbous nasal tip. His palate was high and narrow. Obesity was generalized.

Analysis of the fragile X syndrome gene, *FMR1*, and metabolic screening were normal.

### Patient 2

The second patient is at present 30 years old. His length is 150 cm (-4 1/2 SD), weight is 34 kg (-1 SD) and head circumference 55 cm (-1 1/2 SD). He is severely mentally retarded and is not able to walk or speak. He was born as the third child of non-consanguineous parents after an uneventful pregnancy and delivery. His birth weight was 3000 gram. His muscle tone was weak and developmental delay was obvious within six months. Chromosome analysis in 1980 already showed a translocation with involvement of chromosomes 5, 11 and 13. He had nystagmic eye movements and also epileptic activity, therefore he used antiepileptic drugs. He had sleeping problems, and autistic and self-destructive behaviour (trichotillomania, polyembolokoilamania). Increasingly, he has periods of agitation. He suffers from recurrent ear infections and has almost become blind, at least partially due to automutilation (pushing fingers or other objects in his eyes). He has an asymmetric face with a broad nose and full lips. His right eye is smaller. It is possible that a part of the facial features are the result of the automutilation. There is a highly arched palate with a bifid uvula. Because of the pregnancy of this patient's sister, re-evaluation of his cytogenetic analysis was performed.

## Results

### Patient 1

**Figure 1:**
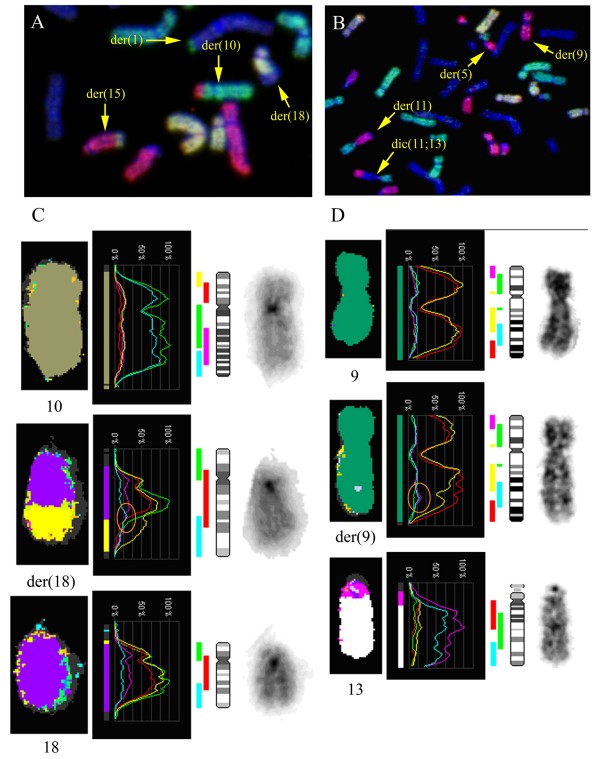
**M-FISH of the aberrant chromosomes of patient 1 A), patient 2 B)**. A partial M-FISH metaphase shows the four derivative chromosomes for both patients. C) The separate fluorochrome signal intensities for the aberrant chromosome in contrast to the normal chromosomes in patient 1 show a slight increase of the blue colour DEAC on the interface between chromosome 18 (combined red, yellow and green signal) and 1 (yellow signal) on der(18) (see orange highlight) which indicates material of chromosome 10 (combined blue and green signal). D) The derivative chromosome 9 (combined red and yellow signal) of patient 2 shows an increase of the combined fluorochromes (blue and purple signals) used for chromosome 13 (see orange highlight), indicating a chromosome 13 insertion. Detection of the insertion of chromosome 5 in der(9) is more difficult with M-FISH, because of the combined colours red and yellow used for chromosome 9 in contrast to red used for chromosome 5, but a more intense fluorescent red signal over yellow is seen in the long arm of der(9). A diminished signal of red and yellow is present on the location of the insertion of chromosome 13.

**Figure 2:**
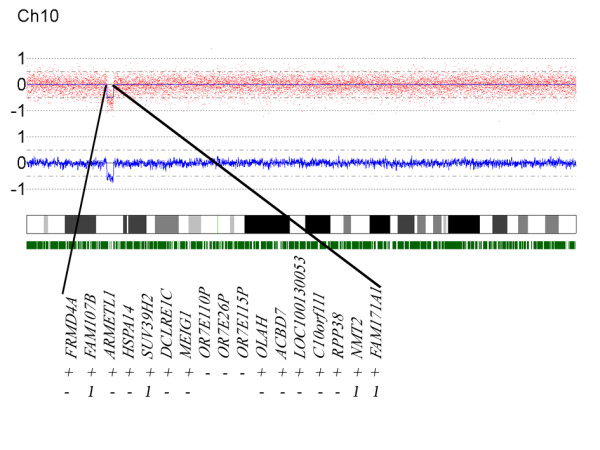
**The interstitial 10p deletion in patient 1 and genes located in this region**. A 250 k Nsp1 SNP-array (Affymetrix) shows a ~1,48 Mb interstitial deletion in chromosome (10)(p13p13) harbouring 17 genes. In the first lane under the genes a + indicates which genes are found in the Ingenuity database. The second lane shows which genes are indirectly correlated to known mental retardation genes presented by the number 1.

**Figure 3:**
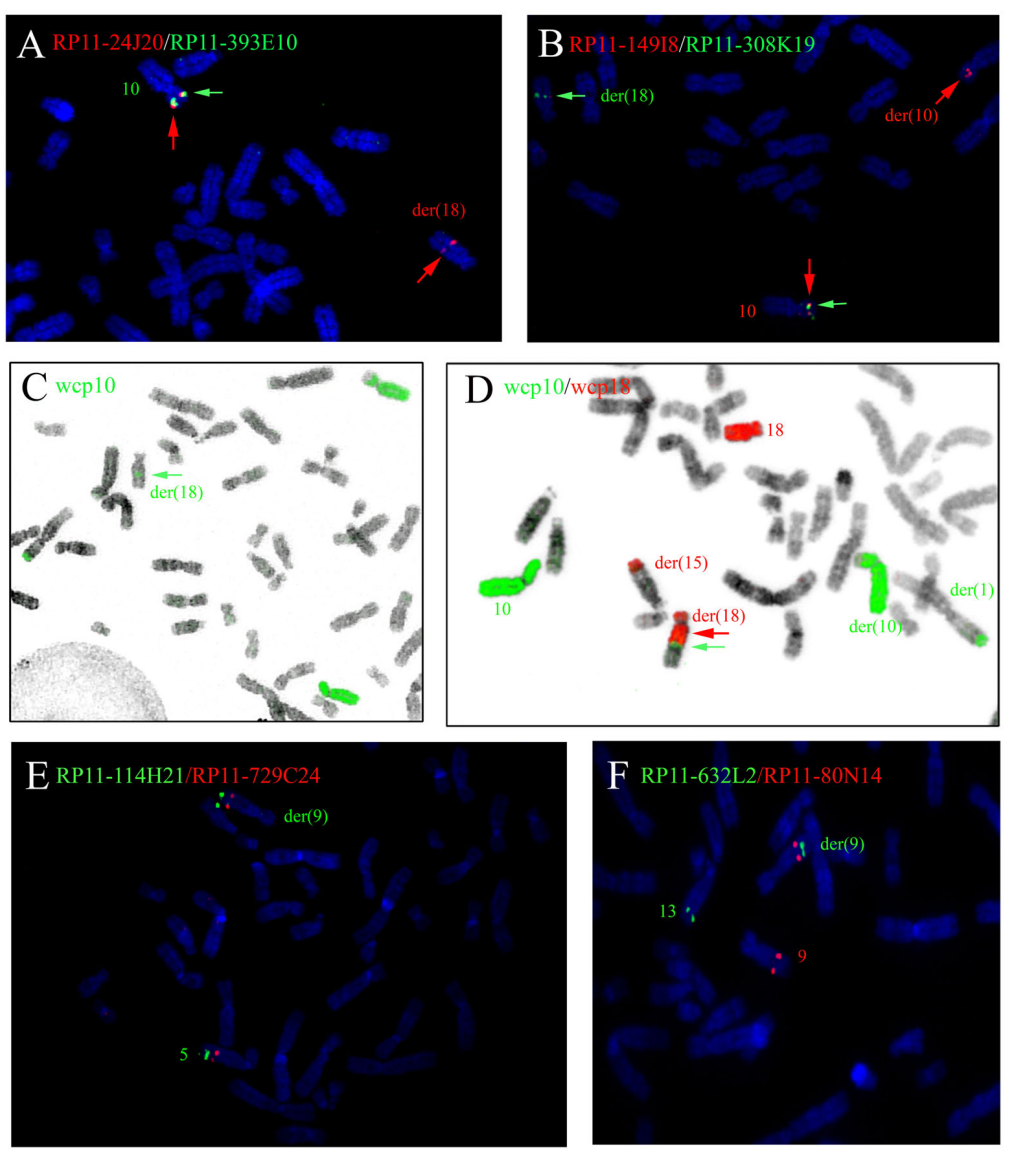
**Detection of the aberrant constitutions of the chromosomes with FISH**. FISH results on patient 1: A) The BAC-probe RP11-393E10 (10p13), green, which is deleted according to the SNP-array results, shows only one signal on the normal chromosome 10, confirming the deletion. RP11-24J20 (10p13), located distal from the deleted region, is present on the normal chromosome 10 and on the der(18). B) Besides present on the normal homologue, RP11-149I8 is located on der(10) and RP11-308K19 on der (18), both overlapping the respectively proximal and distal deletion breakpoints. C) A WCP of chromosome 10 shows four segments distributed over four chromosomes. D) A combined FISH of WCP 18 (red) and WCP 10 (green) shows the presence of chromosome 10 material at the interface of chromosome 18 and chromosome 1 on derivative chromosome 18. FISH results on patient 2: E) BAC-probes RP11-114H21 (green) (5q31.2) and RP11-729C24 (5q31.1) (red) demonstrate a direct insertion in derivative chromosome 9. F) Probe RP11-632L2 (13q31.3) (green) and probe RP11-80N14 (9q31.1) (red) show the insertion of part of chromosome 13 in derivative chromosome 9.

Routine cytogenetic analysis of the patient initially revealed a complex karyotype in which the chromosomes 1, 15 and 18 were involved: 46, XY, t(1;18;15)(q32;q21;q24). Subtelomeric MLPA-analysis showed no copy number changes of the subtelomeric regions (data not shown). M-FISH showed a more complex karyotype in which not only chromosomes 1, 15 and 18, but also chromosome 10 appeared to be involved (Figure 1A). SNP-array analysis revealed an additional interstitial deletion in 10p13, ranging from rs10906541 to rs7911591 (~1,48 Mb) (SNP call of 96.58%; SD 0.257) (Figure 2). The results showed four other copy number changes along the genome, but these were previously reported in healthy individuals in the database of genomic variants as copy number variants (CNVs) [22]. A double-target FISH was performed with regional specific BAC-probes (Table 1). RP11-393E10 (10p13) confirmed the deletion (Figure 3A). The BAC-probe RP11-24J20, located distal to the deletion, demonstrated an unexpected insertion of chromosome 10 in der(18). Also BAC-probe RP11-308K19 was found on der(18) instead of being translocated to chromosome 1 as was expected (Figure 3B). The BAC-probes RP11-308K19 and RP11-149I8, both overlapping the deletion breakpoints in 10p13, coincided with the translocation breakpoints in 10p13, thus confirming the deletion to be related to these breakpoints. FISH with additional BAC-probes located in the 10p13–p14 region (Table 1) identified a direct insertion in 18q21 with the distal breakpoint between RP1-251M9 and RP11-401F24 (data not shown). M-FISH showed a slight increase of the chromosome 10 signal on the interface between the translocated parts of chromosomes 1 and 18 on der(18) (Figure 1C). FISH with individual and combined Whole Chromosome Paints (WCP) confirmed the constitution of the existing derivatives (Figure 3C/D, not all data shown). The results of M-FISH and FISH analysis were used to determine the breakpoints in the derivative chromosomes.

**Table 1:** Overview of characteristics for the BAC-probes used in this study.

**BAC-clone**	**Mb-position (database)**	**Location**	**FISH signal results**	**Origin Probe**
RP5-976H8	9,583339–9,755995 (ensembl)	10p14	10p14 + der(1)	BacPac
RP1-251M9	10,973504–11,104455 (ensembl)	10p14	10p14 + der(1)	BlueGnome
RP11-401F24	11,805219–12,011805 (ensembl)	10p14	10p14 + der(18)	BlueGnome
RP11-477H7	12,396087–12,523522 (ensembl)	10p13	10p13 + der(18)	BlueGnome
RP11-730A19	13,060479–13,254681 (ensembl)	10p13	10p13 + der(18)	BlueGnome
RP11-24J20	13,232015–13,407413 (UCSC)	10p13	10p13 + der(18)	BacPac
RP11-308K19	13,841307–14,026355 (UCSC)	10p13	10p13 + der(18)	BacPac
RP11-393E10	14,421561–14,601389 (UCSC)	10p13	10p13 + Δ	BacPac
RP11-149I8	15,428730–15,540990 (UCSC)	10p13	10p13 + der(10)	BacPac
RP11-32H4	127,185284–127,352319 (UCSC)	5q23.2–23.3	5 + der(11)	BlueGnome
RP11-729C24	131,817004–131,977063 (UCSC)	5q31.1	5q31.1 + der(9)	BlueGnome
RP11-114H21	135,739999–135,916051 (UCSC)	5q31.2	5q31.2 + der(9)	BlueGnome
RP11-433G14	139,529308–139,702096(UCSC)	5q31.3	5q31.3 + dic(11;13)	BlueGnome
RP11-94H11	142,108219–142,285326 (UCSC)	5q31.3	5q31.3 + dic(11;13)	BlueGnome
RP11-436M5	145,784051–145,952287 (UCSC)	5q32	5q32 + dic(11;13)	BlueGnome
RP11-22D7	149,724798–149,897041 (UCSC)	5q33.1	5q33.1 + dic(11;13)	BlueGnome
RP11-26B2	152,539671–152,728895 (UCSC)	5q33.2	5q33.2 + dic(11;13)	BlueGnome
RP11-80N14	105,268990–105,396668 (UCSC)	9q31.1	9 + der(9)	BacPac
RP11-570D4	113,691272–113,781581 (ensembl)	9q31.3	9 + der(9)	BlueGnome
RP11-94M3	90,657469–90,828221 (UCSC)	13q31.3	13q31.3 + dic(11;13)	BacPac
RP11-632L2	92,499761–92,681327 (UCSC)	13q31.3	13q31.3 + der(9)	BlueGnome
RP11-74A12	94,378172–94,507441 (UCSC)	13q32.1	13q32.1 + der(11)	BlueGnome
RP11-79A16	95,390243–95,551839 (UCSC)	13q32.1	13q32.1 + der(11)	BacPac
RP11-813H5	98,714150–98,902701 (UCSC)	13q32.3	13q32.3 + der(11)	BacPac

Parental chromosome and FISH analysis showed normal results.

**Figure 4:**
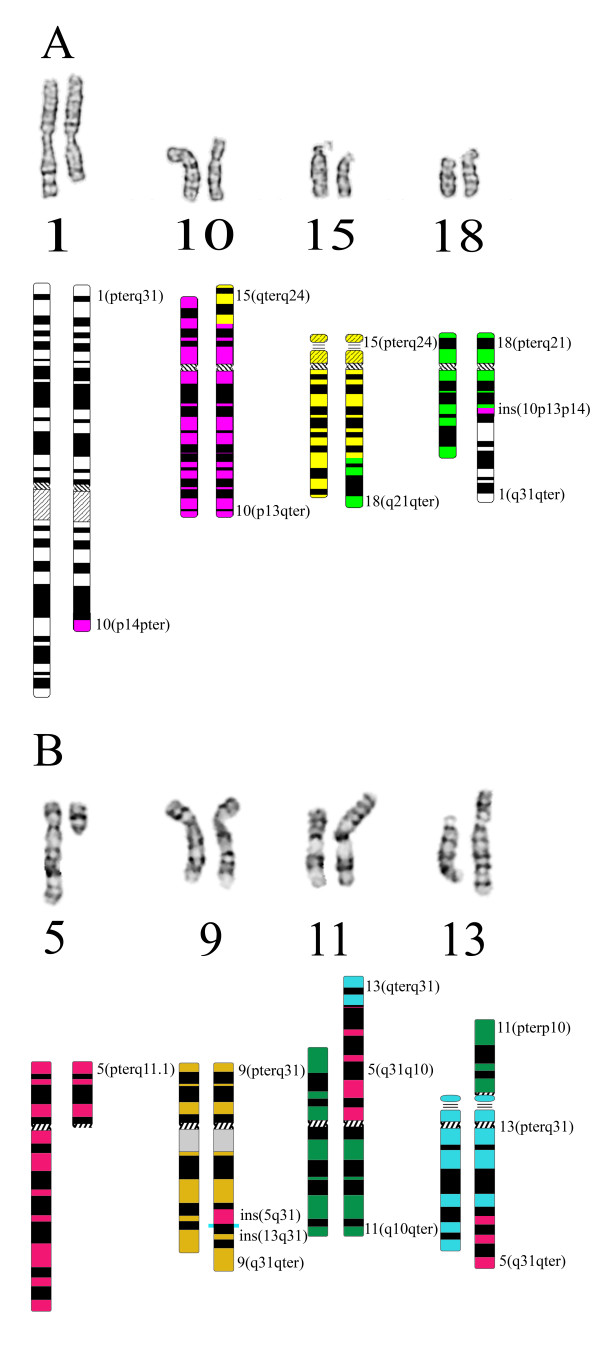
**GTG-banded chromosomes and ideograms**. A partial karyogram accompanied by its ideogram shows the normal (left) and derivative chromosomes (right) which are involved in the complex chromosome rearrangement of patient 1 (A) and patient 2 (B).

The karyotype of patient 1 was readjusted and assigned according to ISCN 2005 [23] as follows:

46, XY, der(1)(1pter→1q31::10p14→10pter), der(10)(15qter→15q24::10p13→10qter)del(10)(p13p13), der(15)(15pter→15q24::18q21→18qter), der(18)(18pter→18q21::10p13→10p14::1q31 →1qter)dn (Figure 4A).

### Patient 2

Conventional banding cytogenetic analysis initially showed a complex karyotype, in which the chromosomes 5, 11 and 13 were involved: 46, XY, del(5)(q11), der(11)t(5;11)(q11;q11), der(13)t(11;13)(q11;p11). M-FISH demonstrated that the chromosomal rearrangement was more complex, and that also chromosome 9 was involved (Figure 1B). FISH with WCPs for chromosomes 5, 9, 11 and 13 and several BAC-probes confirmed the more complex result of the CCR (Table 1). Part of chromosome 5 is inserted in the q-arm of derivative chromosome 9, which was confirmed as a direct insertion with BAC-probes RP11-729C24 and RP11-114H21 (Figure 3E). Also a weak fluorescent signal of WCP 13 was detected on der(9). M-FISH results showed a slight increase of the fluorescent signals for chromosome 13 on der(9) (Figure 1D). FISH with the BAC-probe RP11-632L2 (13q31.3) showed a signal on der(9), confirming the insertion and location of chromosome 13 material into this der(9) (Figure 3F). The insertion of chromosome 5 was located centromeric to the insertion of chromosome 13.

Characterization of the der(13) with several FISH probes revealed that the centromeric probes for chromosome 11 (pLC11A) and 13 (L1.26) were both present on the derivative chromosome 13. Also FISH with the DNA-probe r521 (ribosomal satellite probe) showed that the satellite of chromosome 13 appeared to be located between these two centromeres on the dic(11;13) (data not shown). Subsequent SNP-array analysis showed several small gains and losses, ranging in size from 81 kb till 1 Mb, but all were previously reported as common CNVs in the database of genomic variants (SNP call of 92,98%; SD 0,2035) (data not shown). Since both parents showed normal karyotypes, the karyotype of patient 2 was readjusted and assigned as 46, XY, der(5)(5pter→5p10), der(9)(9pter→9q31::5q31→5q31::13q31→13q31::9q31→9qter), der(11)(13qter→13q31::5q31→5q10::11q10→11qter), dic(11;13) (11pter→11p10::13p13→13q31::5q31→5qter)dn (Figure 4B).

## Discussion

The aim of this study was to characterize the CCRs of two patients with multiple molecular cytogenetic techniques in order to find an explanation for their abnormal phenotype. The application of GTG banding, M-FISH and conventional FISH analysis elucidated the complex chromosomal rearrangements in two patients, each comprising four derivative chromosomes.

In patient 1 the application of a 250 k Nsp1 SNP-array analysis additionally revealed a deletion of part of chromosome 10p13 with an approximate size of 1.5 Mb, harbouring 17 genes. Using the Ingenuity Pathway Analysis program [24], we investigated, whether any of the 17 genes deleted on chromosome 10, could be considered as a candidate gene for mental retardation based on available expression and/or functional data. We found information in the Ingenuity database for 14 of the 17 genes (Figure 2). Four of these genes (*NMT2*, *SUV39H2*, *FAM107B*, *FAM171A1*) showed an indirect relationship with known mental retardation genes of which three genes are expressed in the nervous system (not *SUV39H2*). It is very likely that in patient 1 the *de novo *deletion is causative for his abnormal phenotype, although further examination is necessary to investigate how the deleted genes contribute to his phenotype.

It is known that chromosomal loss of the 10p13–p14 region is associated with DiGeorge syndrome type II with cardiac abnormalities [25]. Yatsenko et al. reported one patient with a larger 10p deletion than our patient has, also including the BAC-probe RP11-393E10 which was absent in patient 1 [26]. Despite this overlap, our patient does not have clinical signs of a congenital heart problem or other symptoms related to the DiGeorge syndrome type II, except for the developmental delay. Christian et al. [27] used array comparative genomic hybridization (array-CGH) to investigate 397 unrelated subjects with autism spectrum disorder. One of the included patients showed a 318 kb deletion on 10p13. However, that deletion was located adjacent to the deletion in our patient, and showed no overlap. To the best of our knowledge, there are no other reports of a correlation of the deleted 10p13 region, or of the other observed breakpoint regions with autism [28-30].

SNP-array analysis of patient 2 showed no additional pathogenic gains or losses with the 250 k Nsp1 platform. FISH revealed a clonal dicentric 11;13 chromosome in all cells. By conventional GTG-banding we observed that the dic(11;13) contained a primary constriction of the centromere 11, suggesting that the centromere 11 is the active centromere and that the centromere 13 is the inactive centromere.

At present, the abnormal phenotype of patient 2 could not be explained by a chromosomal imbalance. In the literature, up to 70% of the patients with a chromosomal rearrangement, both complex and reciprocal translocations, show no imbalance on the chromosomal or molecular level as an explanation for the phenotype [31]. Several other molecular mechanisms have been proposed to explain the clinical problems of these patients [32] such as balanced translocations leading to a position effect by separating a gene from its regulatory elements altering gene-expression [33] or creating fusion genes. A disruption of a gene could unmask a recessive mutation on the homologue allele. Heterochromatin can also have effects on juxtaposed euchromatic regions. This heterochromatinization of euchromatic regions can (partially) silence the expression of neighbouring genes [34]. This might be the case for the der(11) in patient 2, in which the 5q11.2 region might be under the influence of the centromere 11, possibly leading to silencing of important 5q11.2 genes. Finally, also a disruption of a dosage-sensitive gene might alter or eliminate its function, causing disease [35].

As more breakpoints are involved in a CCR such mechanisms as mentioned above suggest a greater chance for an abnormal phenotypic outcome [4]. Madan et al. show that individuals with a CCR and an abnormal phenotype show a significantly higher mean (4.9) of breakpoints than the mean (3.6) of breakpoints for phenotypically normal individuals [3]. In our study the combination of techniques revealed more cryptic rearrangements leading to a total of six breakpoints in patient 1, while patient 2 shows seven breakpoints. Although the deletion in chromosome 10 of patient 1 is assigned as a causative element, DNA rearrangements at the breakpoints could also contribute to the phenotype.

In conclusion, this study demonstrates the power of combining different molecular cytogenetic techniques to elucidate the genetic constitution of CCRs. However, next to M-FISH and high resolution SNP-array analysis, additional FISH analysis with locus specific probes is still crucial to elucidate and identify cryptic genetic abnormalities in more detail, as is demonstrated in this paper. Submicroscopic deletions or duplications will allow further genotype-phenotype correlation studies. On the other hand, the combination of all these molecular cytogenetic analyses does not always explain an abnormal phenotype in patients with a CCR.

## Materials and methods

### Karyotyping

Cytogenetic analysis was performed on GTG-stained metaphase spreads obtained from cultured peripheral blood lymphocytes according to standard procedures. An Axioskop microscope (Zeiss, Sliedrecht, The Netherlands) was used for karyotyping and metaphase images were captured with Ikaros software (Metasystems, Altlussheim, Germany). Karyotypes were obtained from both patients and their parents.

### MLPA

Multiplex Ligation-dependent Probe Amplification (MLPA) was performed using SALSA P036B and P070 kits (MRC Holland, Amsterdam, The Netherlands) to investigate the subtelomeric regions for copy number aberrations according to Schouten et al. (2002) [36]. Analysis was performed using Genemarker^® ^software (SoftGenetics, State College, PA, USA).

### M-FISH

Multicolour FISH was performed using the 24 Xcyte Human mFISH DNA Probe Kit, following manufacturer's instructions (Metasystems). The results were analysed using a Zeiss Imager.Z1 microscope with five filters for the fluorochromes used: diethylaminocoumarine (DEAC), fluorescein isothiocyanate (FITC), SpectrumOrange™, TexasRed™ and Cyanine 5 (Cy™5). The ISIS M-FISH imaging system (Metasystems) was used to capture and process images for evaluation of the M-FISH.

### SNP-Array

A whole genome screening using a high resolution (250 k) Nsp1 SNP-array (Affymetrix, Santa Clara, California, USA) was performed conform manufacturer's specifications. The arrays were scanned using the GeneChip^® ^Scanner 3000 7 G System with autoloader (Affymetrix, Santa Clara, California, USA) and data analysis of the array results was performed using CNAG 3.0 (Copy number analyser for gene chips) provided by . All copy number changes observed were compared to common copy number variants (CNVs) found in previous studies of healthy people annotated in the database of genomic variants (DGV)[37]. Common CNVs are variations of large segments (>1 kb) of the genome that occur in the general public and are assumed to have no clinical significance, i.e. are considered benign CNVs. We looked for CNV studies of appreciable size that were analysed with equal methods.

### FISH

Specific Bacterial Artificial Chromosomes (BAC) probes were selected from the UCSC genome browser (UC Santa Cruz, USA, assembly March 2006) [38] and the Ensembl genome browser (Hinxton, UK, release 52, Dec 2008) [39] and purchased from BACPAC Resourses (Oakland, CA, USA) or from BlueGnome (Cambridge, UK) (Table 1). Whole Chromosome Paint (WCP) probes for chromosomes 1, 5, 9, 10, 11, 13, 15 and 18 (Poseidon, NL) were applied on metaphase spreads according to the manufacturer's specifications.

Probe DNA from BACPAC resources was semi-automatically isolated with an AutoGenPrep 3000 robot (Autogen) and, after whole genome amplification (WGA, Repli-G, Qiagen), digested and labelled (Random Prime labelling system, Invitrogen) with Bio-16-dUTP or Dig-11-dUTP (Roche). BlueGnome probes were provided with direct labels. The probes were validated on control metaphases. FISH experiments were performed according to standard protocols, evaluated on an Axioplan 2 Imaging microscope (Zeiss) and images were captured using Isis software (Metasystems).

## Consent

Written informed consent was obtained from the patients' relatives for publication of this case report. A copy of the written consent is available for review by the Editor-in-Chief of this journal.

## Competing interests

The authors declare that they have no competing interests.

## Authors' contributions

PV drafted the manuscript and participated in the molecular cytogenetic analysis; MS and MD participated in drafting the manuscript on the clinical information; GS, JH, MR and GH carried out (molecular) cytogenetic analyses (respectively GTG-banding, M-FISH, FISH, SNP-array); AV participated in analysing the array results in Ingenuity and bioinformatical databases; PP conceived of the study, and participated in its design and coordination and helped to draft the manuscript. All authors have read and approved the final manuscript.
